# Perspectives on the representation of frailty in the electronic frailty index

**DOI:** 10.1186/s12875-023-02225-z

**Published:** 2024-01-02

**Authors:** Manpreet Thandi, Sabrina T. Wong, Morgan Price, Jennifer Baumbusch

**Affiliations:** 1 School of Nursing, University of British Columbia, T201 2211 Wesbrook Mall, Vancouver, BC V6T 2B5 Canada; 2 Centre for Health Services and Policy Research, University of British Columbia, 201-2206 East Mall, Vancouver, BC V6T 1Z3 Canada; 3 Department of Family Practice, University of British Columbia, David Strangway Building, Suite 300, 5950 University Boulevard, Vancouver, BC V6T 1Z3 Canada

**Keywords:** Frailty, Frailty index, Frailty screening, Aging, Primary care

## Abstract

**Background:**

Frailty is a state of increased vulnerability from physical, social, and cognitive factors resulting in greater risk of negative health-related outcomes and increased healthcare expenditure. A 36-factor electronic frailty index (eFI) developed in the United Kingdom calculates frailty scores using electronic medical record data. There is currently no standardization of frailty screening in Canadian primary care. In order to implement the eFI in a Canadian context, adaptation of the tool is necessary because frailty is represented by different clinical terminologies in the UK and Canada. In considering the promise of implementing an eFI in British Columbia, Canada, we first looked at the content validation of the 36-factor eFI. Our research question was: Does the eFI represent frailty from the perspectives of primary care clinicians and older adults in British Columbia?

**Methods:**

A modified Delphi using three rounds of questionnaires with a panel of 23 experts (five family physicians, five nurse practitioners, five nurses, four allied health professionals, four older adults) reviewed and provided feedback on the 36-factor eFI. These professional groups were chosen because they closely work as interprofessional teams within primary care settings with older adults. Older adults provide real life context and experiences. Questionnaires involved rating the importance of each frailty factor on a 0–10 scale and providing rationale for ratings. Panelists were also given the opportunity to suggest additional factors that ought to be included in the screening tool. Suggested factors were similarly rated in two Delphi rounds.

**Results:**

Thirty-three of the 36 eFI factors achieved consensus (> 80% of panelists provided a rating of ≥ 8). Factors that did not achieve consensus were hypertension, thyroid disorder and peptic ulcer. These factors were perceived as easily treatable or manageable and/or not considered reflective of frailty on their own. Additional factors suggested by panelists that achieved consensus included: cancer, challenges to healthcare access, chronic pain, communication challenges, fecal incontinence, food insecurity, liver failure/cirrhosis, mental health challenges, medication noncompliance, poverty/financial difficulties, race/ethnic disparity, sedentary/low activity levels, and substance use/misuse. There was a 100% retention rate in each of the three Delphi rounds.

**Conclusions and next steps:**

Three key findings emerged from this study: the conceptualization of frailty varied across participants, identification of frailty in community/primary care remains challenging, and social determinants of health affect clinicians’ assessments and perceptions of frailty status. This study will inform the next phase of a broader mixed-method sequential study to build a frailty screening tool that could ultimately become a standard of practice for frailty screening in Canadian primary care. Early detection of frailty can help tailor decision making, frame discussions about goals of care, prevent advancement on the frailty trajectory, and ultimately decrease health expenditures, leading to improved patient and system level outcomes.

**Supplementary Information:**

The online version contains supplementary material available at 10.1186/s12875-023-02225-z.

## Background

The concept of frailty is increasingly recognized in relation to the healthcare of older adults. In 2019, there were over 1.6 million older adults who were identified as frail in Canada [1]. The risk of frailty increases with age. In Canada, individuals aged 65 years and older will make up at least 25% of the population by 2036 [2] totalling 11.9–15.0 million by 2061 [2]. Globally, the proportion of the world’s older adult population is expected to double from 12% in 2015 to 22% in 2050, totalling over two billion people [3].

Frailty is different from the expected physiological decline that occurs with aging because it involves a more rapid decline of stability and abilities, rather than occurring systematically and progressively [4–6]. At an individual level, those living with frailty are more susceptible to greater functional declines in health from illnesses or adverse events considered minor such as the flu or experiencing a fall. They are more likely to be hospitalized, need long-term care, or die as a result of being unable to cope with stressors that would otherwise have minimal impact on healthy individuals [1]. At a system level, increasing frailty is correlated with increased rates of hospitalizations, care home admissions and premature mortality [7–11]. In addition to the implications of frailty on individuals’ physical health and the healthcare system, frailty can significantly impact broader aspects of older adults’ lives. For example, factors such as inactivity, social isolation, loneliness, mental health challenges, and reduced quality of life are also associated with frailty [1, 6]. Conceptualizing frailty beyond its’ physical aspects to include social, emotional, psychological, and environmental contexts that undoubtedly influence individuals’ experiences with frailty will allow for targeted interventions to optimize individuals’ holistic health.

Past research shows that frailty is a relative state and thus is best identified on a continuum since patients can become increasingly frail over time [12–15]. Early identification of frailty and intervention amongst those living in the community can improve individuals’ quality of life and significantly reduce frailty levels and related healthcare system use and expenditure [14, 15]. Identifying frailty in older adults early in primary care was key to the success of interventions identified in previous studies [14, 15] before frailty could no longer be reversed. For example, Theou et al. (2017) [14] identified 14 interventional studies that focused on frailty in older adults living in the community, and 9 of these studies showed that early interventions significantly reduced frailty levels. Additionally, Travers et al.’s (2019) [15] systematic review aimed to assess the effectiveness of 46 studies focusing on frailty interventions in primary care. Fourteen (30%) of these studies reported the outcome of an intervention on frailty status, 71% (*n* = 10) of which demonstrated significant improvement.

Primary care is often the first and main point of contact with the healthcare system for patients and their families, providing an ideal part of the healthcare system to identify and manage frailty. The ongoing nature of primary care clinicians’ relationships with their patients allows them to recognize when individuals are not sufficiently coping with their health and/or social needs [16]. In primary care, the goals of caring for those who are frail are to: 1) prevent or delay increasing frailty severity; 2) improve function and quality of life; and, 3) avoid unnecessary admission to hospital or long-term care [16–18]. Primary care clinicians can manage frailty through tailored and shared decision making about goals of care, preventing advancement on the frailty trajectory, improving health outcomes for older adults, and ultimately decreasing healthcare system expenditures [19].

Yet, accurately and quickly screening for frailty poses a significant challenge for primary care clinicians due to time constraints and competing demands of practice [20]. A recent systematic review reported there are 51 instruments currently used internationally for the detection of frailty but there are no clear suggestions on which ones are most amenable for use in primary care [21]. Key difficulties exist in implementing these instruments within primary care including requiring additional time, difficulty deciding which assessment tool is most appropriate, training of healthcare professionals, use of equipment, and the need for additional clinical resources [12, 22–25]. Although a few existing assessment tools require less than 5–10 min to conduct, this would still be considered an additional task in busy primary care environments. The time burden for primary care clinicians for day-to-day patient care is extremely high and clinicians often have very limited time for activities beyond clinical workflow [26], making existing tools ineffective, unfeasible, and useful for research purposes rather than practice [24]. A screening tool that can automatically calculate frailty scores using existing medical record data mitigates the biggest challenge related to frailty screening in primary care: time.

Additionally, most frailty instruments currently use a binary approach to frailty (frail or not frail) [21] which is less effective for screening purposes in primary care. A binary approach to frailty can lead to frailty being detected too late in an individual’s health trajectory to effectively intervene, manage, reduce, or prevent frailty. When an individual is already identified as frail, they are often too far along on the frailty trajectory to substantially change their health outcomes [15]. Placing frailty on a continuum allows for early identification and appropriate interventions so that frailty can be delayed, reduced, or prevented from becoming worse [13, 14].

A standardized, efficient, and consistent approach is required to case-find those who are frail or at risk of becoming increasingly frail. The broad purpose of this research is to use electronic medical records (EMRs) to automatically populate an electronic frailty screening index and calculate frailty scores for primary care practices’ patient populations. This study is a first step in developing a standardized approach to frailty screening in British Columbia (BC), Canada. The aim of this specific study was to review the conceptualization of frailty in the 36-factor electronic frailty index (eFI) with the intention of ultimately implementing this tool in BC, Canada. To our knowledge, the content of the eFI has not been validated by an interdisciplinary group of healthcare individuals or considered the perspectives of older adults. There is also no tool analogous to the eFI that is used in Canada, and currently no standardization of frailty in Canadian primary care settings. This study aims to address these limitatons.

## Methods

We used a modified Delphi descriptive study research design to answer a specific research question: Does the 36-factor eFI represent the construct of frailty from the perspectives of primary care clinicians and older adults in BC? Our hypothesis was: frailty factors will be suggested for the eFI beyond the ones in the 36-factor eFI. We expected these factors to reflect more contextual aspects of individuals’ lives in addition to the physical and functional factors commonly associated with frailty. This study was approved by the UBC Behavioural Research Ethics Board (H22-00689).

### Electronic frailty index

In this study, frailty is defined as “a state of increased vulnerability from physical, social, and cognitive factors, resulting in a greater risk of negative health-related outcomes including lower quality of life, loss of independence, increased susceptibility to complications, and increased healthcare system utilization” [1, 4–11, 27, 28]. Based on this definition, a promising tool that could be used in primary care to detect frailty using EMR data is the 36-factor electronic frailty index (eFI) (herein, the 36-factor eFI). The eFI is a standard of practice across the United Kingdom (UK) and is shown to demonstrate good construct and predictive validity in predicting mortality, hospitalizations, and nursing home admissions for increasingly frail patients [12]. It automatically calculates a frailty score from EMR data, mitigating the requirement for additional clinician time related to completing a frailty assessment. The 36-factor eFI is an acceptable and feasible starting point in addressing frailty; it shows significant promise in enabling individualized and comprehensive care planning, targeting of interventions, improving health service planning, and facilitating patient-centered care for older people with complex health issues [22, 29–31]. Adaptation of the eFI to a Canadian context is necessary because different clinical terminologies are used in Canadian primary care settings compared to the UK.

### The Delphi method

The Delphi process is a methodological approach used to identify the collective opinion of individuals who are experts in their fields [32] as a valid approach to group consensus. An expert is defined as an “individual with relevant knowledge and experience of a particular topic” [25], p.120]. Hsu & Sandford [32] and Sekayi & Kennedy [33] state that consensus on a topic in a Delphi method can be confirmed if a certain percentage of the votes fall within a prescribed range, notably 70–80%.

Compared to a traditional Delphi process, the modified Delphi allows for both quantitative and qualitative data collection, analysis, and interpretation of data. It also allows for a range of question types beyond the traditional Likert scale questions common to the traditional Delphi. In this study, the modified Delphi allowed for rating scale questions, and open ended responses which were key in understanding the rationale behind ratings for frailty factors and additional factors suggested for inclusion in a frailty screening tool. The modified Delphi also provided opportunities for participants to reflect on their responses and provide feedback after each round to ensure their perspectives and ideas were valued and included throughout the research process.

The Delphi method is recommended for use in healthcare research as a reliable way to determine consensus for a defined research problem [34–37]. It assumes that the judgements of a group of people are more valid than individual judgements [34–36] and that the expertise of panelists is more important than the number [38]. While Eubank et al. [35] state that 5–10 panelists are considered adequate, most Delphi studies have used between 15–20 panelists [33] to achieve heterogeneity of views and content validation. We aimed to recruit 15–20 participants in order for the group size to contribute meaningful data while still being manageable to coordinate. We also aimed for equal representation of the various groups of participants (primary care physicians, nurse practitioners, nurses, allied health professionals, older adults) to prevent overrespresentation of any one specific group and to enhance the the diversity of knowledge sources and experiences that can inform the conceptualization of frailty.

### Delphi process as guided by Hohmann et al. [39]

Table 1 shows steps outlined by Hohmann et al. (2018) [39] for carrying out a modified Delphi. Communication between researchers remained open for panelists to ask any questions or concerns they had throughout this process. The multiple iterations and feedback processes allowed panelists to re-examine their initial judgements in a reflective and anonymous way. The purpose of open-ended responses was to allow panelists to reflect on other participants’ ratings of frailty factors to inform their own thinking and conclusions. Table 1 lists the nine steps used to examine the 36 eFI frailty factors. The modified Delphi took approximately 16 weeks, occurring between July 2022 and November 2022.

**Table 1:** Steps in the modified Delphi approach used to examine frailty

Step 1	Defining the problem and developing related questions
Step 2	Selection of diverse expert panel
Step 3	Distribution of the questionnaire to the panel: Round 1
Step 4	Analysis and summary of the data and development of follow up questionnaires
Step 5	Repeat above steps (3 and 4): Rounds 2 and 3
Step 6	Panelists invited to review consensus and specify reasons for dissenting opinion: throughout all 3 rounds
Step 7	Repeat steps 4–6 if needed
Step 8	Summary of consensus and provision of feedback to the panel
Step 9	Final consensus document developed and distributed

#### Step 1: Defining the problem and developing the questionnaire

The defined problem consists of two main issues: (1) the factors representing frailty in the 36-factor eFI needed content validation, and (2) the selection of frailty factors in the 36-factor eFI did not account for the experiences of diverse primary care clinicians or older adults. Participants were provided with two peer-reviewed articles [28, 40] and one policy statement from Doctors of BC [41] as background readings before beginning the questionnaires. All three resources discussed the need to conceptualize frailty holistically in addition to its biomedical aspects. Clegg et al.’s [12] article highlighting the development of the 36-factor eFI was also provided as an optional resource for participants to review.

A questionnaire was developed to allow participants to rate each frailty factor and suggest additional factors to include in a screening tool for frailty. The questionnaire provided a structured and systematic approach to achieve consensus on frailty factors to be used in primary care frailty screening. The questionnaire was developed specifically for this study and is available as Supplemental material.

#### Step 2: Selection of diverse expert panel (eligibility criteria)

Eligible participants were recruited from pre-existing relationships within the British Columbia Canadian Primary Care Sentinel Surveillance Network (BC-CPCSSN) [42] during May–June 2022. We used purposive, convenience sampling to recruit primary care physicians, nurse practitioners, nurses, other allied health professionals and patients via email. These professional groups were targeted for recruitment because they work together as close interprofessional teams within primary care settings to optimize the health of their patients, a large number of which are older adults. Older adults were targeted for recruitment because they can provide real life experiences and context regarding their health trajectories. Recruitment emails were sent by MT (first listed author). Participants were given two weeks to respond to the invitation to participate in the study. Follow-up emails were sent one week and ten days after the initial recruitment email. After two weeks, weekly follow-up emails were sent to those we had not heard from. If there was no response after one month, the assumption was they were unable to participate.

Clinician participants (physicians, nurse practitioners, nurses, allied health) were included in the study if they:

- were practicing clinicians in a primary care clinic with older adults for at least 5 years; or,
- have previous experience working in primary care with older adults for at least 5 years; or,
- were currently practicing in primary care with at least 5 years total years of clinical experience working with older adults.

Clinician participants were excluded if:

- they did not provide longitudinal care or had limited experience with community-based care (physicians and nurse practitioners only)

Older adults were 65 years or older and needed to have an interest in frailty. Interest in frailty was assessed during introduction meetings when participants were required to indicate their interest in helping with the study topic.

#### Step 3: Distribution of the questionnaire to the panel: round 1 (procedures)

Individual meetings, over Zoom, were held to explain the study, what was expected of each participant, and to obtain informed consent. Panelists were emailed the questionnaire link via Qualtrics online survey software. Panelists rated the importance of each frailty factor by assigning it a score out of 10 (0 = no relevance to frailty/should not be included; 10 = highly relevant to frailty/should be included). Based on past work [17], if a score of less than 8 was assigned to a frailty factor, panelists were asked to provide open-ended responses to explain their rationale. In the first questionnaire, panelists also had the opportunity to suggest additional frailty factors that they believe should be included in the eFI and why (open-ended responses).

Panelists were given two weeks to complete the questionnaire and were sent two reminders to do so, one week prior to the deadline, and three days prior to the deadline. The reminders were sent via email via Qualtrics, and if there was still no response, panelists who did not respond were individually followed up with via email. Panelists did not have the option to leave any blanks in the questionnaires.

#### Step 4–7: Analysis and summary of the data and development of follow up questionnaires (analysis)

Iterative analyses occurred throughout the Delphi process. Quantitative analysis was used to determine the level of agreement for each frailty factor. To achieve consensus on a frailty factor, 80% agreement (i.e., 19 of the 23 participants rating the factor as eight or above) was required. If a factor achieved consensus, it was removed from the subsequent questionnaire. Qualitative analysis included summarizing the open-ended responses. We created reports summarizing the questionnaires and sent them to panelists for their review before each new Delphi round. Additional factors were reviewed and categorized by similarity based on the clinical judgement of the research team. A table was created listing each of the panelists’ suggestions, where the suggestion was placed, and the rationale for its placement (see Supplemental material). Suggestions were also compared against the original eFI frailty factors and associated codes. The factors that were determined to already be incorporated within the eFI were not presented in the subsequent questionnaire for panelists to rate.

#### Step 8: Summary of consensus and provision of feedback to the panel & Step 9: final consensus document developed and distributed

Panelists were given the opportunity to comment on a report that was developed summarizing all Delphi rounds. This final report included clinical definitions of the 36 frailty factors that were reviewed as well as the explanations provided by panelists for the additional suggested factors.

Descriptive statistics of study participants were also gathered including numbers of participants, clinical backgrounds, years of experience, age, sex, and education levels.

## Results

This study had a 100% response rate for Delphi rounds (*n* = 3) from all 23 participants.

### Participants

Participants included 19 clinicians (five family physicians, five nurse practitioners, five nurses, two social workers, one clinical counsellor, and one clinical pharmacist). Four older adults participated; three were patients of three different clinicians and one was the relative of another older adult. The mean age for clinians was 46 years, ranging from 29–66 years old; the mean age for older adults was 71 years, ranging from 65–80 years old. The clinicians’ years of experience ranged from 5–40 years with a mean of 17.9 years. At least one of the physicians, nurse practitioners, and primary care nurses were currently practicing in a primary care setting. All allied health team members were currently practicing in primary care.

Table 2 summarizes the descriptive characteristics of study participants.

**Table 2:** Demographic characteristics (*n* = 23)

**Demographic Data**	
Total number of panelists	23
Age: mean (SD; range)	51 (14; 29–80)
Clinicians (*n*=19)	46 (12; 29–66)
Older Adults (*n*=4)	71 (6; 65–80)
Sex	
Female: *n* (%)	18 (78)
Clinicians (*n*=19)	15 (79)
Older Adults (*n*=4)	3 (75)
Clinicians’ (*n* = 19) Clinical background, *n* (%)	
Physician	5 (22)
Nurse Practitioner	5 (22)
Nurse	5 (22)
Allied healthcare professional	4 (17)
Clinicians’ (*n* = 19) Years of Clinical Experience, *n* (%)	
5–10	7 (37)
> 10	12 (63)
Clinicians’ years of experience: mean (SD; range)	17.9 (12.6; 5–40)
Education level (highest completion level): *n* (%)	
Post-secondary degree	11 (48)
Clinicians (*n*=19)	8 (42)
Older Adults (*n*=4)	3 (75)
Graduate degree	12 (52)
Clinicians (*n*=19)	11 (58)
Older Adults (*n*=4)	1 (25)

### Delphi rounds – eFI factors

A total of 33 of 36 (92%) eFI factors achieved consensus from panelists (Table 3). There were three frailty factors that did not achieve consensus after all three rounds: hypertension, peptic ulcer, and thyroid disorder. Stated reasons by participants for rating these three factors below eight included:

- These conditions on their own do not cause frailty
- They are easily treated, managed, and/or controlled
- They are not determining factors in frailty

Some participants who rated these factors below eight did not provide reasons as to why.

**Table 3:** Summary table of eFI frailty factors achieving consensus

Factors that achieved consensus after round 1 (*n* = 12)	Additional factors that achieved consensus after round 2 (*n* = 17)	Additional factors that achieved consensus after round 3 (*n* = 4)	Factors that did not achieve consensus (n = 3)
1. Fragility Fracture	1. Arthritis	1. Atrial Fibrillation	1. Hypertension
2. Heart Failure	2. Chronic Kidney Disease	2. Urinary System Disease	2. Peptic Ulcer
3. Parkinson’s Disease	3. Coronary Heart Disease	3. Hearing Loss	3. Thyroid Disorder
4. Stroke/TIA	4. Diabetes	4. Anaemia and Haematinic Deficiency	
5. Dyspnoea	5. Foot Problems		
6. Falls	6. Heart Valve Disease		
7. Memory and/or cognitive impairment	7. Hypotension/Syncope		
8. Urinary Incontinence	8. Osteoporosis		
9. Activity Limitation	9. Peripheral Vascular Disease		
10. Housebound	10. Respiratory Disease		
11. Mobility and/or Transfer Problems	11. Skin Ulcer		
12. Requirement for Care	12. Dizziness		
	13. Weight Loss and/or Anorexia		
	14. Polypharmacy		
	15. Sleep Disturbances		
	16. Social Vulnerability		
	17. Vision Problems/Blindness		

Table 4. summarizes rating scores and which round each of the frailty factors achieved consensus. Sources for clinical definitions were the Centers for Disease Control and Prevention (CDC) [43], National Institutes of Health (NIH) [44], Diabetes Canada [45], World Health Organization (WHO) [46], Mayo Clinic [47], National Kidney Foundation (NKF) [48], National Health Service (NHS) [49], National Cancer Institute (NCI) [50], Healthline [51], National Eating Disorders Association (NEDA) [52], and the Heart and Stroke Foundation (HSF) [53].

**Table 4:** Frailty factors achieving consensus (*n* = 33)

Frailty Factor	Clinical Definition	Rating *Round: Mean (SD)*	Consensus (Rating ≥ 8) *Round, n (%)*
Activity Limitation	Difficulties in performing tasks and engaging in social roles [43]	Round 1: 7.91 (2.04)	Round 1, 19 (83)
Anaemia and Haematinic Deficiency	Anaemia is a condition that develops when your blood produces a lower than normal amount of healthy red blood cells. Haematinics are nutrients required for the formation of blood cells (i.e. iron, B12, folate) [44]	Round 1: 7.00 (1.78)	Round 3, 22 (96)
		Round 2: 7.48 (1.41)	
		Round 3: 8.13 (0.55)	
Arthritis	Inflammation or swelling of one or more joints. It describes conditions that affect the joints, tissues around the joints, and other connective tissues [43]	Round 1: 7.14 (2.10)	Round 2, 21 (91)
		Round 2: 8.04 (1.07)	
Atrial Fibrillation	The most common type of treated heart arrythmia. An arrythmia is when the heart beats too slowly, too fast, or in an irregular way [43]	Round 1: 6.13 (1.98)	Round 3, 20 (87)
		Round 2: 7.52 (1.44)	
		Round 3: 8.04 (0.71)	
Cerebrovascular Disease	Stroke/TIA (mini-stroke); an acute compromise to the blood flow through the brain [44]	Round 1: 8.52 (1.78)	Round 1, 20 (87)
Chronic Kidney Disease	A condition characterized by gradual loss of kidney function over time; the kidneys are damaged and cannot filter blood as well they should, resulting in the buildup of excess fluid and waste products [43, 48]	Round 1: 7.74 (1.54)	Round 2, 21 (91)
		Round 2: 8.52 (1.08)	
Coronary Heart Disease	Type of heart disease that occurs when the arteries of the heart (coronary arteries) cannot deliver enough oxygen-rich blood to the heart, often caused by a plaque buildup in the walls of the coronary arteries [43, 44]	Round 1: 8.00 (1.31)	Round 2, 23 (100)
		Round 2: 8.65 (0.78)	
Diabetes	A disease that occurs when one’s blood glucose/sugar is high because their body either can’t produce insulin or can’t properly use the insulin it produces [45]	Round 1: 7.30 (1.66)	Round 2, 20 (87)
		Round 2: 8.26 (1.10)	
Dizziness	A term used to describe a range of sensations such as feeling faint, weak, lightheaded, or unsteady [47]	Round 1: 7.65 (1.97)	Round 2, 21 (91)
		Round 2: 8.26 (1.54)	
Dyspnoea	Sensation of difficult or uncomfortable breathing/shortness of breath [44]	Round 1: 8.13 (2.07)	Round 1, 20 (87)
Falls	An event which results in a person coming to rest inadvertently on the ground or floor or other lower level [46]	Round 1: 9.09 (1.53)	Round 1, 20 (87)
Foot Problems	Issues related to feet	Round 1: 7.39 (1.85)	Round 2, 21 (91)
		Round 2: 8.13 (1.42)	
Fragility Fracture	Any fracture that occurs from trauma [44]	Round 1: 8.52 (1.44)	Round 1, 19 (83)
Hearing Impairment	Inability to hear as well as someone with normal hearing [46]	Round 1: 6.87 (2.58)	Round 3, 22 (96)
		Round 2: 7.91 (1.0)	
		Round 3: 8.43 (0.84)	
Heart Failure	Heart fails to pump sufficient levels of blood and oxygen to support other organs in the body [43]	Round 1: 8.39 (1.08)	Round 1, 20 (87)
Heart Valve Disease	When any valve in the heart has damage or is diseased and/or the heart valves do not open or close properly [43, 53]	Round 1: 7.83 (1.37)	Round 2, 20 (87)
		Round 2: 8.22 (1.28)	
Housebound	Unable to leave one’s home, often due to illness or functional/mobility limitation	Round 1: 8.48 (1.97)	Round 1, 19 (83)
Hypotension/Syncope	Low blood pressure; develops when blood flows through the arteries at higher-than-normal pressures (syncope = fainting as a result) [44]	Round 1: 7.91 (2.15)	Round 2, 21 (91)
		Round 2: 8.39 (1.47)	
Memory and/or Cognitive Problems	Issues with conscious intellectual activity such as thinking, reasoning, or remembering [47]	Round 1: 8.78 (1.44)	Round 1, 19 (83)
Mobility and Transfer Problems	Difficulties getting around physically or moving from one place to another [47]	Round 1: 8.96 (1.02)	Round 1, 22 (96)
Osteoporosis	Condition that weakens bones, making them more fragile and more likely to break [49]	Round 1: 8.13 (1.22)	Round 2, 20 (87)
		Round 2: 8.35 (1.27)	
Parkinsonism and Tremor	Brain disorder that causes unintended or uncontrollable movements such as shaking, stiffness, and difficulty with balance and coordination [44]	Round 1: 8.65 (1.03)	Round 1, 20 (87)
Peripheral Vascular Disease	Blood circulation disorder that affects blood vessels outside the heart and brain [43, 51]	Round 1: 7.35 (1.67)	Round 2, 20 (87)
		Round 2: 8.26 (0.81)	
Polypharmacy	The use of multiple medications simultaneously [44]	Round 1: 7.96 (2.08)	Round 2, 21 (91)
		Round 2: 8.48 (1.12)	
Requirement for Care	Requiring help from others for personal care	Round 1: 8.91 (1.08)	Round 1, 21 (91)
Respiratory Disease	Disease that affects the lungs and other parts of the respiratory system, affecting breathing [50]	Round 1: 8.09 (1.16)	Round 2, 22 (96)
		Round 2: 8.91 (0.85)	
Skin Ulcer	An open sore caused by poor blood flow [51]	Round 1: 7.26 (1.96)	Round 2, 20 (87)
		Round 2: 7.91 (0.95)	
Sleep Disturbance	Disorders of initiating and/or maintaining sleep, disorders of excessive sleeping, disorders of sleep–wake schedule, and dysfunctions associated with sleep, sleep stages, or partial arousals [44]	Round 1: 6.83 (1.90)	Round 2, 20 (87)
		Round 2: 7.83 (1.40)	
Social Vulnerability	Potential negative effects on communities caused by external stresses on human health [43]	Round 1: 8.13 (1.60)	Round 2, 23 (100)
		Round 2: 9.04 (0.82)	
Urinary Incontinence	Loss of bladder control, unintentional passing of urine [44, 47]	Round 1: 7.57 (2.27)	Round 1, 19 (83)
Urinary System Disease	Diseases or disorders that affect the urinary system [44]	Round 1: 6.71 (1.95)^a^	Round 3, 20 (87)
		Round 2: 7.78 (1.00)	
		Round 3: 8.22 (0.80)	
Visual Impairment	Inability to see as well as someone with normal vision [46]	Round 1: 7.74 (2.12)	Round 2, 22 (96%)
		Round 2: 8.87 (0.87)	
Weight Loss and/or Anorexia	Losing weight faster than normal (anorexia – eating disorder caused by weight loss [52]	Round 1: 7.74 (2.20)	Round 2, 22 (96)
		Round 2: 8.52 (1.68)	

^a^Urinary System Disease Round 1: One panelist rated this factor as 6 for males and 8 for females. Another panelist rated this factor as 7 for bladder problems, 1 for polyps, and 9 for incontinence. This mean and standard deviation for “urinary system disease” does not include these 2 participants’ ratings

Table 5 summarizes how the factors that did not achieve conensus were rated in each round of the Delphi. The source for clinical definitions was the National Institutes of Health (NIH) [44].

**Table 5:** Frailty factors not achieving consensus (*n* = 3)

Frailty Factor	Clinical Definition	Rating *Round: Mean (SD)*	(Rating ≥ 8) *Round, n (%)*
Hypertension	High blood pressure; develops when blood flows through the arteries at higher-than-normal pressures [44]	Round 1: 6.17 (2.44)	Round 1, 9 (39)
		Round 2: 7.39 (1.70)	Round 2, 14 (61)
		Round 3: 7.74 (1.45)	Round 3, 17 (74)
Peptic Ulcer	Sores to stomach/small intestine lining [44]	Round 1: 5.78 (2.33)	Round 1, 8 (35)
		Round 2: 6.45 (1.87)^a^	Round 2, 10 (43)
		Round 3: 7.26 (1.42)	Round 3, 13 (57)
Thyroid Disease	Condition that keeps the thyroid from producing the right amount of hormones (i.e. too much or too little) [44]	Round 1: 5.13 (2.36)	Round 1, 4 (17)
		Round 2: 5.87 (1.98)	Round 2, 8 (35)
		Round 3: 6.74 (1.54)	Round 3, 13 (57)

^a^Peptic Ulcer Round 2: One panelist rated this factor as “either 7 or 9”: This mean and standard deviation does not include this participant’s ratings

### Delphi rounds – additional factors

Table 6 lists the additional factors suggested by panelists and summarizes ratings for each of the suggested factors in rounds 2 and 3 of the Delphi. There were a total of 13 suggested factors that were rated by panelists across the 2^nd^ and 3^rd^ questionnaires. Twelve of these factors achieved consensus; “race/ethnic disparity” did not. 

Additional suggested factors were rated on 2 occasions: in round 2 and 3 (if needed) of the Delphi.

**Table 6:** Additional factors suggested with definitions

Frailty Factor	Panelists’ Definitions	Rating *Round: Mean (SD)*	Consensus (Rating ≥ 8) *Round, n (%)*
Cancer	- It may be the only issue a person has and it could be terminal	Round 2: 8.43 (1.31)	Round 2, 20 (87)
Challenges to Healthcare Access	- Inconsistency in finding and accessing medical services	Round 2: 8.52 (1.04)	Round 2, 21 (91)
	- Lack of transportation		
	- Anyone without a health care advocate (meaning someone to accompany to appointments, pick up prescriptions, wellness checks etc.)		
	- Physical isolation (living far out, no access to transport)		
	- No primary care provider/lack of family doctor		
	- Access to regular health care provider		
	- Lack of a family doctor		
Chronic Pain/Back Pain	- Chronic pain / back pain	Round 2: 8.30 (1.15)	Round 2, 20 (87)
	- Pain		
Communication Challenges *(rated a 2*^*nd*^* time to clarify definition)*	- inability to communicate verbally-not able to advocate for themselves, needs, desires for ongoing health care and goals of care	Round 2: 8.04 (1.26)	Round 2, 19 (83)
		Round 3: 8.48 (1.31)	Round 3, 21 (91)
	- Handwriting deterioration		
	- Language/cultural barriers		
	- Low literacy		
Fecal Incontinence *(new category created post-reflection after 1*^*st*^* round)*	Fecal Incontinence (a sign of failure of other organs)	Round 3: 8.65 (0.93)	Round 3, 21 (91)
Food Insecurity	- Noticeable lack of interest in meal preparation and meal planning	Round 2: 7.96 (1.19)	Round 2, 19 (83)
	- Reduction in amount of fruit and vegetables in diet		
	- Sense of taste and smell diminish		
	- Food insecurity		
Liver Failure/Cirrhosis	Liver failure/cirrhosis (end-organ failure)	Round 2: 8.13 (1.29)	Round 2, 19 (83)
Mental Health Challenges	- Profound mental illness that impacts day to day functioning	Round 2: 8.65 (0.93)	Round 2, 22 (96)
	- Trauma/history of trauma		
	- Profound mental Illness (i.e. schizophrenia) that impacts day to day functioning		
	- Depression		
	- Anxiety		
	- Mood disorders		
	- Mental illness/mental health disorders		
	- Psychosis		
Medication Noncompliance *(new category created post-reflection after 1*^*st*^* round)*	Noncompliance to medication (can impact an individuals mental and physical health depending on what the medication is used for)	Round 3: 8.39 (1.47)	Round 3, 22 (96)
Poverty/Financial Difficulties *(new category created post-reflection after 1*^*st*^* round)*	- Poverty (inability to gain access to resources and services required to prevent health decline)	Round 3: 9.04 (0.93)	Round 3, 23 (100)
	- Financial difficulties (leaving people unable to afford basic necessities)		
	- Loss of income- inability to afford medication, health equipment, housing		
	- Low income		
	- Low socioeconomic status; these individuals may not have access to adequate nutrition, transportation to appointments or the means to pay out of pocket for prescriptions necessary to optimize health		
	- Financial insecurity		
Race/Ethnicity Disparity *(new category created post-reflection after 1*^*st*^* round)*	Race/ethnicity disparity (i.e. differences in health/health outcomes between racial/ethnic groups)	Round 3: 7.70 (1.49)	Consensus not achieved
Sedentary/Low Activity Levels	- Sedentary/low activity levels	Round 2: 8.09 (1.12)	Round 2, 21 (91)
	- Views on exercise and activity		
Substance Use/Misuse	- Substance misuse/abuse—alcohol in particular in the elderly but other recreational drug abuse as well	Round 2: 8.35 (1.27)	Round 2, 21 (91)
	- Alcohol/Smoking		
	- Addiction		
	- Drug and substance use disorders		

## Discussion

This study addresses content/face validity of the 36-factor eFI with a panel of experts that included clinicians and older adults, aged 65 years and older. Almost all the eFI factors were highly rated by the panelists. Additionally, the majority of the new suggested factors encompassed more than biomedical diagnoses and functional limitations: challenges to healthcare access, communication challenges, food insecurity, mental health challenges, medication noncompliance, poverty/financial difficulties, race/ethnic disparities, and substance use/misuse. Not surprisingly, findings demonstrated that frailty is a complex phenomenon involving individuals’ social, emotional, psychological, and environmental contexts [28].

### Context matters

Current conceptualizations of frailty tend to be informed by evidence that arises from biomedical theories and do not often account for the living or environmental conditions that contribute to increasing frailty. However, there is growing literature recognizing the impact of broader social and contextual factors that influences one’s frailty status. For example, people’s experiences with what gets labelled as frailty are shaped by various intersecting factors; these include but are not limited to age, gender, social class, social environment, societal perceptions, geography, life experiences, and several other social determinants of health [28, 54, 55]. A key limitation of the 36-factor eFI is the focus on disease states. Of the 36 deficits listed in the eFI, 20 are placed in the disease state category, and of the remaining 16, it can be argued that only one (social vulnerability) considers the broader context of patients’ lives. The eFI, like many frailty assessment tools, is largely reflective of biomedicine in its focus on deficits and clinical signs and symptoms, rather than functional capabilities and contextual factors [22]. This study demonstrated that several panelists recognized this limitation in the 36-factor eFI and reflected on the importance of social determinants of health and contextual factors that might affect an individual’s frailty status. Due to the emphasis on biomedical billing codes in primary care, it can be challenging to integrate these broader factors into a tool that uses electronic data to screen for frailty. However, extracting free text from electronic medical records may capture social, emotional, pyshcological, and environmental factors that are often missed when using clinical terminology systems that largely focus on biomedical billing codes. Our subsequent study will explore this possibility. Additionally, although beyond the scope of our study, future work can consider revising the eFI to include the additional factors suggested by participants in this study.

Research shows an increased risk of frailty in the presence of eight of the new suggested factors: cancer [56]; chronic pain [57–59]; fecal incontinence [60, 61]; liver failure/cirrhosis [62, 63]; mental health challenges [64–67]; poverty/financial challenges [68, 69]; race/ethnic disparity [70]; and sedentary/low activity levels [71–73]. Research shows mixed results and/or a bi-directional relationship between frailty and two of the suggested factors: food insecurity [74–79] and medication noncompliance [80, 81]. Further research is required looking at the relationship between frailty and three of the suggested factors: challenges to healthcare access, communication challenges, and substance use/misuse.

### The conceptualization of frailty varies across participants

Current approaches to frailty prioritize mostly physicians’ and researchers’ knowledge. Our study suggests additional healthcare providers such as nurse practitioners, nurses, and allied health have vast experience in working with older adults and can provide valuable input to the development of a primary care frailty screening tool. Additionally, older adults’ perspectives are not often included in the development of tools that are meant to benefit them, even though they can provide valuable subjective knowledge. This study addressed these gaps in the 36-factor eFI.

Panelists agreed that diagnoses and functional limitations are important to realize when assessing for frailty as evident by 33 of the 36 eFI factors achieving consensus after three Delphi rounds. There were differences in opinion about some factors that were perceived to be easily treatable or manageable (hypertension, thyroid disorder, and peptic ulcer did not achieve consensus).

The diversity of the group of primary care clinicians and older adults constituting the expert panel contributes to the credibility of this work and may also contribute to the differences in responses. The differences in ratings of frailty factors speaks to the subjective nature of frailty conceptualization. Individuals often have different ideas about what frailty is and how it should be defined. Noteably, older adults’ ratings didn’t appear to noticeably differ from clinicians’ ratings. Older adults drew on their own experiences in considering whether the factors indicated someone was frail. For example, if they experienced one of the factors (i.e. hypertension, arthritis, diabetes, etc.), they did not necessarily view themselves as frail. Moreover, if a condition was treatable, it was not viewed as a contributing factor to frailty. For example, incontinence, hearing impairment, and polypharmacy were seen as problems with solutions (i.e. incontinence pads, hearing aids, managing medications). It was also stated by two of the older adults that frailty is attitudinal and the result of psychological problems and outcomes that can be modified with behavioural and lifestyle changes. These findings highlight the need to include older adults as partners in future research and in collaborative care planning. Future research related to tool development should similarly consider the perspectives of both the individuals who will be using the tool (i.e., clinicians) as well as the individuals the tool is meant to benefit (i.e., older adults).

### Identification of frailty in community/primary care remains challenging

The complex nature of frailty often leads to key challenges associated with its’ identification and management [19]. Although primary care clinicians are well placed to identify and manage frailty [16, 18], there is currently no standardized data collection tool for frailty in North America. There are key difficulties with implementing existing frailty tools in primary care such as requiring additional time, difficulty deciding which tool is most appropriate, training of healthcare professionals, use of equipment and the need for additional resources [12, 22–25]. There is a gold standard that exists for frailty assessment – the Comprehensive Geriatric Assessment (CGA) [82]. However, this often takes 30–60 min to complete and thus is not feasible to conduct on every patient that accesses primary care services. The time burden for primary care clinicians for day-to-day patient care is already high and clinicians often have very limited time for activities beyond clinical workflow [26]. For these reasons, there is significant potential to use EMRs to collect data and screen for frailty.

Additionally, frailty is difficult to identify because it is not a construct that is directly diagnosable with specific biomedical markers such as blood pressure readings for hypertension or blood sugar levels for diabetes. Frailty is complex and needs to be considered in the context of individuals’ broader life circumstances. A case-finding approach needs to be implemented [83] in order to (1) screen for individuals who require comprehensive follow up (with the CGA for example), and (2) develop holistic and individualized care plans to manage frailty.

### Impact on future frailty research and clinical practice

The aim of our broader research is to use EMRs to automatically populate an electronic frailty screening index and calculate frailty scores for primary care practices’ patient populations, through adaptation of the existing eFI. This current study was a first step towards this goal, to first validate the content of the tool. We summarize four significant impacts of this work on future frailty research and clinical practice.

1. Enhancing the use of primary care EMRs and potentially artificial intelligence methods to automatically assign patients a frailty score to determine whether follow up is needed.
2. Mitigating the biggest change in frailty screening and assessment: time.
3. Recognizing frailty on a continuum and recognizing factors beyond the biomedical that could be incorporated into maintaining (or even improving) individuals’ health status.
4. The potential of the eFI becoming a standard of practice in BC primary care settings.

## Limitations

Although the expert Delphi panel was interprofessional and included older adults, the sample only included individuals from BC. Future similar studies could include primary care clinicians and older adults from other regions in BC or other provinces in Canada, depending on the target region of tool implementation, in order to enhance generalizability of the findings. Doing so may also reveal whether our findings might differ if the study was conducted in other geo-cultural contexts. Informal caregivers such as family members were not included in this study; future research should consider the perspectives of these key individuals as they spend significant amounts of time with their loved ones and may provide valuable knowledge about factors that influence frailty. Participants were also volunteers who likely have a greater interest in frailty research than those who did not volunteer to participate in the study.

The study was cost-effective as it was done virtually; however, multiple reminders were needed for participants to complete the questionnaires. The purpose of a frailty screening tool and how it works needed to be explained to participants regularly to address participants’ reasons for rating specific factors below 8. For example, participants stated certain conditions were not determining factors in frailty because they can be easily treated/controlled and these conditions on their own don’t cause frailty. Participants needed to be reminded that frailty is additive of multiple factors and that no one factor, by itself, makes someone frail. Additionally, it is difficult to know whether a condition is in fact being treated or managed without screening for it first by including it in a screening tool.

## Conclusions

This work resulted in a review of the 36-factor eFI as well as 13 additional suggested factors that were perceived to represent frailty. Three key findings emerged from this study. (1) Context matters: there was general agreement that the eFI screening tool is missing key elements of individuals’ holistic health, emphasizing that frailty needs to be conceptualized as more than just biomedical diagnoses and functional limitations. (2) The conceptualization of frailty varied across participants, indicating that there is a subjective component contributing to how individuals understand frailty. (3) The identification of frailty in primary care remains challenging, indicating a need for a standardized case-finding approach to initiate frailty prevention and management. As part of a broader three-part research study, the 36 frailty factors will be mapped to standardized clinical terminologies to inform the development of an algorithm that will provide frailty scores for patients in BC primary care practices. Additional frailty factors suggested by participants in this study will also be considered for future work beyond this study to encourage a more holistic approach to frailty.

Having a standardized method to to screen for frailty using already collected EMR data could potentially improve both primary care clinical practice and coordination of care with community based organizations, while alleviating the challenges related to the identification and management of frailty in community and/or primary care settings. This research also aligns with policy directions from the BC Ministry of Health in which frailty initiatives prioritize early detection, intervention, and management services to enhance quality of care and improve healthcare system spending [83]. The proposed study shows promise in enabling the targeting of interventions, improving health service planning, and facilitating continuous, comprehensive, and patient-centered primary care [12].

### Supplementary Information

**Additional file 1.** 

## Data Availability

All data generated or analyzed during this study are included in this published article (and its supplementary information file). Any further requested information is available from the corresponding author on reasonable request.

## Abbreviations

eFI: Electronic Frailty Index
EMR: Electronic Medical Record
BC: British Columbia
UK: United Kingdom
BC-CPCSSN: British Columbia Canadian Primary Care Sentinel Surveillance Network
CDC: Centers for Disease Control and Prevention
NIH: National Institutes of Health
WHO: World Health Organization
NKF: National Kidney Foundation
NHS: National Health Service
NCI: National Cancer Institute
NEDA: National Eating Disorders Association
HSF: Heart and Stroke Foundation
CGA: Comprehensive Geriatric Assessment
