# Supplementary material for: Perspectives on the representation of frailty in the electronic frailty index
Source: BMC Prim Care. 2024 Jan 2;25:4. doi: 10.1186/s12875-023-02225-z (PMC10759446; doi:10.1186/s12875-023-02225-z)
Supplement: Supplementary file 1 — Additional file 1. [file 12875_2023_2225_MOESM1_ESM.zip › 12875_2023_2225_MOESM1_ESM.docx]

**Additional Factors Suggested by Delphi Panelists**

Delphi panelists were given the opportunity to suggest factors in addition to the 36 included in the 36-factor eFI. The suggested factors were reviewed and categorized based on the clinical judgement of the research team. The below table lists each of the panelists’ suggestions, where the suggestion was placed, and the rationale for its placement. This activity resulted in a total of 13 additional suggested factors that panelists then rated in rounds 2 and 3 of the Delphi.

**Supplemental Table: Additional Factors Suggested and Rationale for Placement**

| **Factor Suggested** | **Placement** | **Why** |
| --- | --- | --- |
| Poverty (inability to gain access to resources and services required to prevent health decline) | Additional category 🡪 Poverty/Financial Difficulties | There is no current category that encompasses poverty; perhaps “vulnerable adult”? |
| Housing (state of house, safety risks, rugs for falls) | Social Vulnerability | Already included under social vulnerability under “housing unsatisfactory” |
| Quality of family connections and support | Social Vulnerability | Already included under social vulnerability under “social problem” |
| Trauma | Additional category 🡪 mental health challenges | History of trauma is known to affect long term mental health |
| Depression and Profound Mental Illness (i.e. schizophrenia) | Additional category 🡪 mental health challenges | No current category that encompasses this |
| Financial difficulties (I would consider to be different than social (which is certainly quite encompassing) - people with poor finances who cannot afford basic necessities are at much higher risk of functional decline. | Additional category 🡪 Poverty/Financial Difficulties | There is no current category that encompasses poverty and/or low income/financial difficulties; perhaps “vulnerable adult”? |
| Sedentary/low activity levels - associated with sarcopenia and deconditioning and further frailty. | Additional category 🡪 Sedentary/Low Activity Levels | No current category that encompasses this |
| Mood disorders - whether depression or anxiety or psychotic disorders, mood disorders are often associated with worsening function and frailty and cognitive decline | Additional Category 🡪 mental health challenges | No current category that encompasses this |
| Fecal Incontinence (often associated with frailty and functional decline, and a sign of failure of other organs) | Additional Category 🡪 Fecal Incontinence | No current category that encompasses this |
| Liver failure/cirrhosis (end-organ failure, highly associated with frailty) | Additional Category 🡪 Liver Failure/Cirrhosis | No current category that encompasses this |
| Substance misuse/abuse - alcohol in particular in the elderly but other recreational drug abuse as well. | Additional Category 🡪 Substance Misuse/Abuse | No current category that encompasses this |
| Severe mental health challenges- which impacts day to day functioning | Additional Category 🡪 mental health challenges | No current category that encompasses this |
| Noncompliance to medication (can impact an individuals mental and physical health depending on what the medication is used for) | Additional Category 🡪 medication noncompliance | No current category that encompasses this |
| loss of income- inability to afford medication, health equipment, housing | Additional category 🡪 Poverty/Financial Difficulties | There is no current category that encompasses poverty; perhaps “vulnerable adult”? |
| inability to communicate verbally-not able to advocate for themselves, needs, desires for ongoing health care and goals of care | Additional Category 🡪 communication challenges | No current category that encompasses this |
| Loss of housing- no shelter to hold personal items or get a good nights rest or means to care for themselves | Social Vulnerability | Already included under social vulnerability under “housing unsatisfactory” |
| Inconsistency in finding and accessing medical services | Additional 🡪 Challenges to healthcare access | No current category that encompasses this |
| Lives alone and needs care | Social Vulnerability | Already included under social vulnerability under “Lives alone/no help available” |
| Alcohol/Smoking | Additional Category 🡪 Substance Misuse/Abuse | No current category that encompasses this |
| Social Isolation/Loneliness | Social Vulnerability | Already included under “social problem” |
| Mood disorders | Additional Category 🡪 mental health challenges | No current category that encompasses this |
| Cognitive Impairment | Memory and Cognitive Problems | Already encompassed in this category |
| Anticoagulant Medication Use (prone to bleed/hospitalization easily) | Not included | Not specific enough |
| Increasing social disengagement. Relationship with family and peers, attendance at social functions or parties. Entertains company far less. | Social Vulnerability | Already included under “social problem” code |
| Apathy. Marked decrease in interests and engagement in community and world affairs. From engagement to observer to disinterest. Interests in life-held hobbies/interests diminishes | Social Vulnerability | Already included under “social problem” code |
| Noticeable lack of interest in meal preparation and meal planning | Additional Category 🡪 Food Insecurity | No current category that encompasses this |
| Handwriting deterioration | Additional Category 🡪 communication challenges | No current category that encompasses this |
| Reduction in amount of fruit and vegetables in diet | Additional Category 🡪 Food Insecurity | No current category that encompasses this |
| Walks much more slowly | Mobility and Transfer Problems | Already encompassed in this category |
| Balance is much more unsteady | Mobility and Transfer Problems | Already encompassed in this category |
| Sense of taste and smell diminish | Additional Category 🡪 Food Insecurity | No current category that encompasses this |
| Dry eyes | Visual Impairment | Already a code “Referral to ophthalmology service” which would occur if dry eyes was a significant issue |
| Drops off to sleep while sitting up during the day | Additional category 🡪 Sedentary/low activity levels | No current category that encompasses this |
| Experiences lessening of digital 'tactile-ness'…and fine motor co ordination | Requirement for Care | Already encompassed in this category; this would result in needing help from others |
| Self 'pedicare' ability diminishes..toes and toenails are a challenge | Foot Problems | Already encompassed in this category |
| Increasing dependence on others for bathing, housework, shopping, banking | Requirement for Care | Already encompassed in this category |
| Increased unreliability in managing finances | Requirement for Care | Already encompassed in this category |
| Critical capacities seem to diminish leaving one much more vulnerable and gullible to frauds and scam | Social Vulnerability / Memory and Cognitive Problems | Already encompassed in these categories |
| Recent loss of spouse | Social Vulnerability | Already encompassed in this category |
| Lack of transportation | Additional 🡪 Challenges to healthcare access | No current category that encompasses this |
| Low income | Additional category 🡪 Poverty/Financial Difficulties | There is no current category that encompasses poverty; perhaps “vulnerable adult”? |
| Lives alone | Social Vulnerability | Already included under social vulnerability under “Lives alone” |
| No family/informal caregivers | Social Vulnerability | Already encompassed in this category |
| Proximity to caregiver family members | Social Vulnerability | Already encompassed in this category |
| Language barriers | Additional Category 🡪 communication challenges | No current category that encompasses this |
| Mental illness | Additional Category 🡪 mental health challenges | No current category that encompasses this |
| Addiction | Additional Category 🡪 Substance Misuse/Abuse | No current category that encompasses this |
| External or Informal Support system; individuals without close family members or friends, who are socially isolated I would consider highly vulnerable. | Social Vulnerability | Already encompassed in this category |
| Anyone without a health care advocate (meaning someone to accompany to appointments, pick up prescriptions, wellness checks etc) is at the mercy of our health care system. If this factor is combined with a serious health condition I would consider that individual as experiencing frailty. | Additional 🡪 Challenges to healthcare access | No current category that encompasses this |
| Low socioeconomic status; these individuals may not have access to adequate nutrition, transportation to appointments or the means to pay out of pocket for prescriptions necessary to optimize health. In combination with other factors could be considered frailty. | Additional category 🡪 Poverty/Financial Difficulties | There is no current category that encompasses poverty; perhaps “vulnerable adult”? |
| Chronic pain / back pain | Additional Category 🡪 Chronic pain/back pain | No current category that encompasses this |
| Views on exercise and activity- If as a young person they didn't find benefit in physical activity, that will carry into adulthood and older adulthood | Additional category 🡪 Sedentary/Low Activity Levels | No current category that encompasses this |
| Physical isolation (living far out, no access to transport) | Additional 🡪 Challenges to healthcare access | No current category that encompasses this |
| Mental health- depression, and anxiety could be crippling | Additional Category 🡪 mental health challenges | No current category that encompasses this |
| Pain | Additional Category 🡪 Chronic pain/back pain | No current category that encompasses this |
| Dependence/reliance on others for help | Requirement for Care | Already encompassed in this category |
| Social support system | Social Vulnerability | Already encompassed in this category |
| Resources (financial, housing.. | None | Already encompassed in other categories depending on the resource |
| Living Alone | Social Vulnerability | Already included under social vulnerability under “Lives alone” |
| Diabetes- on insulin /Sulfonylureas Multiple morbidities ( ie 3+) | Diabetes | Already encompassed in this category |
| Language barrier with co morbid conditions | Additional Category 🡪 communication challenges | No current category that encompasses this |
| No primary care provider | Additional 🡪 Challenges to healthcare access | No current category that encompasses this |
| Financial insecurity | Additional category 🡪 Poverty/Financial Difficulties | There is no current category that encompasses poverty; perhaps “vulnerable adult”? |
| Food insecurity | Additional Category 🡪 Food Insecurity | No current category that encompasses this |
| Drug and substance use disorders, | Additional Category 🡪 Substance Misuse/Abuse | No current category that encompasses this |
| Hx. trauma, | Additional category 🡪 mental health challenges | History of trauma is known to affect long term mental health |
| Mental health disorders | Additional Category 🡪 mental health challenges | No current category that encompasses this |
| Access to regular health care provider | Additional 🡪 Challenges to healthcare access | No current category that encompasses this |
| Race/ethnicity disparity | Additional Category – Race/Ethnicity Disparity | No current category that encompasses this |
| Self reported performance on how they function at home (do they ned help on a regular basis/have help if they need it? ) – | Not included | Unable to extract this data from EMRs, some of these factors are covered elsewhere |
| Language/cultural barriers? – | Additional Category 🡪 communication challenges | No current category that encompasses this |
| Mental health? | Additional Category 🡪 mental health challenges | No current category that encompasses this |
| Poverty | Additional category 🡪 Poverty/Financial Difficulties | There is no current category that encompasses poverty; perhaps “vulnerable adult”? |
| Lack of a family doctor | Additional 🡪 Challenges to healthcare access | No current category that encompasses this |
| Lack of social supports | Social Vulnerability | Already encompassed in this category |
| Low literacy | Additional Category 🡪 communication challenges | No current category that encompasses this |
| Inadequate footwear | Foot Problems | Already encompassed in this category |
| Mental health or lack thereof | Additional Category 🡪 mental health challenges | No current category that encompasses this |
| Depression | Additional Category 🡪 mental health challenges | No current category that encompasses this |
| Psychosis | Additional Category 🡪 mental health challenges | No current category that encompasses this |
| Cancer wasn’t included. It may be the only issue a person has and it could be terminal | Additional Category 🡪 Cancer | No current category that encompasses this |

**Legend:**

| Factor is already included within the eFI |
| --- |
| Added as a new “additional suggested factor” |
| Not included (i.e. irrelevant, unable to code, etc.) |
