# Supplementary material for: Perspectives on the representation of frailty in the electronic frailty index
Source: BMC Prim Care. 2024 Jan 2;25:4. doi: 10.1186/s12875-023-02225-z (PMC10759446; doi:10.1186/s12875-023-02225-z)
Supplement: Supplementary file 1 — Additional file 1. [file 12875_2023_2225_MOESM1_ESM.zip › 12875_2023_2225_MOESM2_ESM.docx]

**Delphi Questionnaire – Round #1**

*Note: this questionnaire will be distributed through Qualtrics Online Survey Software.*

Definition of **frailty** used in this research:

A state of increased vulnerability from physical, social, and cognitive factors, resulting in a greater risk of negative health-related outcomes including lower quality of life, loss of independence, increased susceptibility to complications, and increased healthcare system utilization.

Please rate the importance (by assigning it a score out of 10) of each of the following factors according to how relevant you feel they are to reflecting the construct of frailty. If you choose to rate a factor below 8/10, please explain why.

Arthritis /10

Atrial Fibrillation *(irregular heart rhythm)*  /10

Chronic Kidney Disease /10

Coronary Heart Disease /10

Diabetes /10

Foot Problems /10

Fragility Fracture *(fracture resulting from a fall)* /10

Heart Failure *(heart does not pump enough blood)* /10

Heart Valve Disease /10

Hypertension *(high blood pressure)* /10

Hypotension/Syncope *(low blood pressure/fainting)* /10

Osteoporosis *(weakened bones)* /10

Parkinson’s Disease /10

Peptic Ulcer *(sores on stomach/small intestine lining)* /10

Peripheral Vascular Disease *(blood circulation disorder)* /10

Respiratory Disease /10

Skin Ulcer /10

Stroke/TIA /10

Thyroid Disorder /10

Urinary System Disease /10

Dizziness /10

Dyspnoea *(shortness of breath)* /10

Falls /10

Memory and/or Cognitive

Problems /10

Weight Loss and/or Anorexia /10

Polypharmacy *(use of 5 or more medications)* /10

Sleep Disturbance /10

Urinary Incontinence *(loss of bladder control)* /10

Activity Limitation /10

Hearing Loss /10

Housebound /10

Mobility and Transfer Problems /10

Requirement for Care /10

Social Vulnerability /10

Vision Problems/Blindness /10

Anaemia & Haematinic Deficiency /10

*(low red blood cells/oxygen)*

Are there additional factors not listed above that you think should be included to reflect frailty? We encourage you to think beyond biomedical factors and consider all aspects of individuals’ lives. Please list additional factors below and explain why you think they should be included to reflect frailty.

*Note: The subsequent questionnaires (#2 and #3) will include factors that did not achieve consensus (i.e., did not achieve 80% agreement) and panelists will follow the same instructions they did for this questionnaire. Panelists will no longer have the opportunity to suggest additional frailty factors after the first questionnaire, but the suggested factors will be similarly rated in rounds 2 and 3.*
